# Programming Crystallographic Orientation in Additive‐Manufactured Beta‐Type Titanium Alloy

**DOI:** 10.1002/advs.202302884

**Published:** 2023-07-28

**Authors:** Xuan Luo, Tao Song, Annett Gebert, Kai Neufeld, Ivan Kaban, Hongwei Ma, Weisi Cai, Haizhou Lu, Dongdong Li, Ning Li, Yuanyuan Li, Chao Yang

**Affiliations:** ^1^ National Engineering Research Center of Near‐net‐shape Forming for Metallic Materials Guangdong Provincial Key Laboratory for Processing and Forming of Advanced Metallic Materials South China University of Technology Guangzhou 510640 China; ^2^ Institute for Complex Materials Leibniz IFW Dresden 01069 Dresden Germany; ^3^ State Key Laboratory of Materials Processing and Die and Mould Technology Huazhong University of Science and Technology Wuhan 430074 China

**Keywords:** additive manufacturing, crystallographic orientation, directional solidification, properties

## Abstract

Additively manufactured metallic materials typically exhibit preferential <001> or <110> crystallographic orientations along the build direction. Nowadays, the challenge is to program crystallographic orientation along arbitrary 3D direction in additive‐manufactured materials. In this work, it is established a technique of multitrack coupled directional solidification (MTCDS) to program the <001> crystallographic orientation along an arbitrary 3D direction in biomedical beta‐type Ti‐Nb‐Zr‐Ta alloys via laser powder bed fusion (LPBF). MTCDS can be achieved via directional solidification of coupled multi‐track melt pools with a specific temperature gradient direction. This results in continuous epitaxial growth of the β‐Ti phase and consequently sets the <001> crystallographic orientation along an arbitrary 3D direction. This way, relatively low elastic modulus values of approximately 60 ± 1.2 GPa are customized along an arbitrary 3D direction. It is expected that MTCDS can be generalized to a wide range of applications for programming specific crystallographic orientations and, respectively, tailoring desired properties of different metallic materials.

## Graphical abstract

A technique of multi‐track coupled directional solidification (MTCDS) to program the <001> orientation along an arbitrary 3D direction is established in additively manufactured β‐Ti alloys. MTCDS can be achieved via directional solidification of coupled multi‐track melt pools with a specific temperature gradient direction. This technology provides a method to develop biomedical Ti alloys with low elastic modulus and other high‐performance metallic materials.

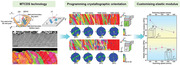

## 1 Introduction

Additive manufacturing (AM) of metals, such as laser powder bed fusion (LPBF), enables the fabrication of near‐net shape metallic components with complex geometries via track‐by‐track and layer‐by‐layer rapid solidification by varying process parameters including power, scanning speed, hatch spaces, and scanning strategies.^[^ ^1^, ^2^, ^3^, ^4^ ^]^ The fusion‐based metal AM process is based on spatial stacking of micro‐areas solidified from the melt pool,^[^ ^1^, ^5^ ^]^ integrating a complex solidification process involving epitaxial growth of crystals, solute distribution, and melt convection. Variation of process parameters and thus the crystallization conditions inside moving melt pool and spatial stacking of solidified micro‐areas enable to diversify the crystallographic orientations. For example, changing the overlapping style between layers by varying scan strategies results in a transition of the preferred orientation from <011> to <001> along the building direction. ^[^ ^6^, ^7^, ^8^ ^]^ Changing the scanning speed induces two orientations, <011> and <001>, along the building direction.^[^ ^9^ ^]^ Although AM is mainly regarded as a programmable forming technology in terms of microstructure design and resultant macroscopic performance, researchers have not yet taken full advantage of its capabilities for obtaining desired macroscopic performance of fabricated metallic materials by tailoring the spatial stacking of solidified micro‐areas and associated metallurgical processes.^[^ ^1^, ^10^, ^11^, ^12^ ^]^

It has been shown that temperature gradients inside solidifying micro‐areas are the key factor governing the epitaxial growth direction of crystals.^[^ ^1^, ^17^, ^18^, ^19^ ^]^ This can result in preferred orientations in metallic materials, such as the <001> crystallographic orientation in duplex stainless steels during gas arc welding.^[^ ^20^ ^]^ The epitaxial growth of crystals is spatially variable with the movement of melt pools during AM, and depends on the spatial and temporal variations of the temperature gradient. This affects the local curvature of the solidification interface inside the melt pools.^[^ ^18^, ^21^ ^]^ As a result, the AM process is prone to locally produce an ordinary microstructure without a preferred crystallographic orientation, in addition to the typical columnar grain microstructure with <001> and <011> orientations along the building direction.^[^ ^22^, ^23^, ^24^ ^]^ Essentially, the temperature gradient of the melt pool boundary is approximately constant and identical in a specific spatial range or time period at the initial solidification stage of the melt pool.^[^ ^18^, ^21^, ^25^, ^26^ ^]^ It is normal to the solidifying interface of the melt pool,^[^ ^27^, ^28^, ^29^ ^]^ determining the growth direction of the crystals. As such, coupling remelting of a previous melt track, epitaxial growth of crystals between melt tracks, and directional solidification along a specific direction can be obtained during the entire AM process, producing the preferred crystallographic orientation and desired performance of additively manufactured metallic materials, specifically a low elastic modulus along an arbitrary 3D direction. This is particularly interesting for the fabrication of bone implants. Novel beta‐type titanium alloys exhibit low Young's modulus values beneficial for relieving the stress‐shielding effects. The next challenge is to generate tailored microstructural states yielding low elastic modulus values along a specific 3D direction, in accordance with the anisotropic mechanical properties of long bones.^[^ ^6^, ^13^ ^]^ Theoretically, this can be achieved by programming the <001> crystallographic orientation during additive manufacturing.^[^ ^6^, ^14^, ^15^, ^16^ ^]^

In this study, we established a methodology of multi‐track coupled directional solidification (MTCDS) for programming crystallographic orientations in additive manufacturing, specifically LPBF. This was achieved by precisely coupling multi‐track melt pools with a tailored temperature gradient direction, resulting in directional solidification and epitaxial growth. This enabled to obtain additively manufactured metallic parts with <001> crystallographic orientation along arbitrary spatial direction. We chose the beta‐type Ti‐35Nb‐7Zr‐5Ta (wt.%) alloy, which has the potential for biomedical use, to demonstrate the validity of the MTCDS methodology. We anticipate that this methodology can be applied for additive manufacturing of high‐performance metallic materials for different applications.

## 2 Results

### 2.1 Multi‐track Coupled Directional Solidification (MTCDS)

**Figure 1:**
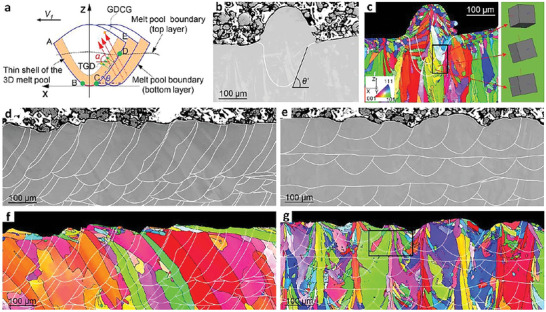
a) Schematic of MTCDS technique. Points B and E are the intersections between the top and bottom layers. Points B and C are the intersections between the left and right adjacent melt pools. The melt pool angle *θ* is the angle between line CD and the horizontal direction. The columnar grain angle *α* is the angle between the GDCG and the horizontal direction. The TGD is normal to the melt pool boundary and approximately normal to line CD. b, c) SEM and EBSD images of single melt‐track. d,e) SEM micrographs and f,g) corresponding EBSD images of LPBF‐fabricated samples produced at hatch distances of 40 µm and 120 µm with the same laser power (250 W) and scanning speed (600 mm s^−1^). The side wall angle *θ'* is the angle between the melt pool boundary and the horizontal direction, which is similar to *θ*.

**Figure** **1** shows a schematic and selected morphologies demonstrating the principles of the MTCDS technique. The diagram in Figure 1a presents directional solidification along a specific growth direction of the columnar grain (GDCG) by precisely coupling multi‐track melt pools. The solid–liquid interface of the melt pool, which moves during AM process, has a thin‐shell 3D region (light‐orange in Figure 1a) in its front at the initial solidification stage. The CD sector of this region has an almost unchanged temperature gradient direction (TGD), ensuring constant growth direction of the columnar grains.^[^ ^25^, ^26^ ^]^ Herein, we used the unchanged TGD inside the 3D regions in multiple melt tracks to guarantee directional solidification. This results in successive epitaxial growth of columnar grains located at the melt pool boundary with the same curvature, which is defined as the multi‐track coupled directional solidification (MTCDS) technique. Specifically, the 3D region has three types of columnar grains in three sectors, AB, BC, and CE, corresponding to three different curvatures of the melt pools, in which columnar grains have the same and unchanged growth directions. The curvature of the CD surface can be considered as constant, thereby facilitating directional solidification in multiple melt tracks by epitaxial growth. To prove the existence of the described 3D region with directional solidification, Figure 1b,c shows the scanning electron microscope (SEM) and electron backscatter diffraction (EBSD) images of the single melt track. The transverse cross‐section of the single melt track has a specific domain that consists of multiple columnar grains growing epitaxially from pre‐existing polycrystals in the substrate. The crystal axes of multiple columnar grains in the specific domain are almost parallel to each other (unit cell wireframe in Figure 1c) and are not perpendicular to the melt pool boundary. Multiple columnar grains between the two white lines in the framed region (Figure 1c) grow in the preferential direction of preexisting polycrystals, which is dominated by an almost parallel temperature gradient. This indicates that a specific region is capable of maintaining directional solidification.

To further verify the validity of MTCDS, Figure 1d–g shows the SEM microstructures and corresponding EBSD images of the LPBF samples prepared with optimized hatch distances. As shown in Figure 1d,f, MTCDS can be realized at an extremely low hatch distance of 40 µm. As the right sector of the 3D melt pool is stacked in parallel (Figure 1a), the epitaxial growth of multiple melt tracks can proceed continuously in an almost fixed direction, referred to as directional solidification in this study. In essence, this is attributed to two extremely high overlap rates, ≈66.4% and 80% for the n and n‐1 tracks and the n and n+1 tracks (n is an even number), respectively. The former and latter correspond to the thicker and thinner halves of the 3D melt pool (Figure 1d). The high overlap rates result in survival of the BC and CE sectors of the melt pool, as shown in Figure 1c. In contrast, MTCDS could not arise at a relatively high hatch distance of 120 µm (Figure 1e,g) due to the low overlap rate (approximately 22.0%). In such a case, the curvature of the BC sector varies over a wider range, resulting in survival of columnar grains with nearly symmetrical crystal orientations (black box in Figure 1g). At the same laser power (250 W) and scanning speed (600 mm s^−1^), the shape and size of the melt pools were obviously different, as shown in Figure 1d,e. A similar scenario was observed for LPBF‐processed Inconel 718.^[^ ^30^ ^]^ This resulted from differences in the energy absorption efficiency and the temperature field at different hatch distances, which are determined by different denudation extents of metal powder layers driven by the Bernoulli effect.^[^ ^31^, ^32^ ^]^ Based on the results of the single‐track and multi‐track experiments, the MTCDS methodology can be established.

### 2.2 Crystallographic Orientation Variation with Melt Pool Angle

**Figure 2:**
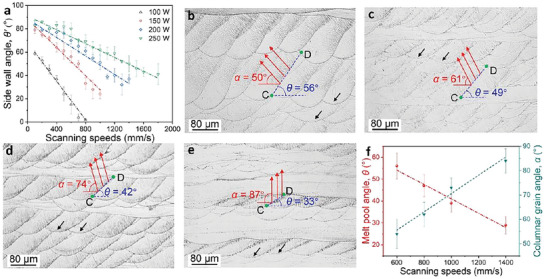
a) Sidewall angle *θ'* as a function of scanning speed at different laser powers. b–e) OM images of cross‐sections of LPBF specimens processed with different scanning speeds: b) 600 mm s^−1^; c) 800 mm s^−1^; d) 1000 mm s^−1^; e) 1400 mm s^−1^. f) Variation of melt pool angle (*θ*) and columnar crystal angle (*α*) upon change of the scanning speed.

In the established MTCDS methodology, it is essential to determine the growth direction of columnar grains (GDCGs). In general, the crystallographic orientation is affected by the geometry of the melt pool, which in turn is influenced by the LPBF parameters and material properties.^[^ ^1^, ^20^, ^33^ ^]^ **Figure** **2**a shows the geometrical evolution of the melt pool in dependence on the processing parameters, in terms of the sidewall angle *θ'* as function of the scanning speed. It is found that the value of *θ'* decreases linearly with an increase in the scanning speed, accompanied by an increased inclination angle (approximately equal to 90°‐*θ'*) between the TGD and the horizontal direction. The growth direction aligns with the TGD as much as possible.^[^ ^18^ ^]^ As such, the dependence of *θ'* on the laser power and scanning speed (Figure 2a) provides the possibility of tailoring the solidification direction and the resultant crystallographic orientation, as in the MTCDS diagram in Figure 1a. Evidently, the higher the power, the smaller the slope of the linear variation, as shown in Figure 2a. This indicates that it is easier to locate a specific *θ'* and the associated direction of solidification. Thus, we produced LPBF samples with different crystallographic orientations by varying the scanning speed at a fixed power of 250 W (Figure 2b–e). Regular stacking between adjacent melt tracks often maintains continuous epitaxial growth of grains in all samples. The CD sector at every melt‐pool boundary is nearly parallel, which results from the parallel TGDs between adjacent melt pools and the resultant directional solidification. The grain growth direction related to the melt‐pool geometry gradually turns to the building direction with an increase in scanning speed.

Considering the different solidification environments of the single‐track and multi‐track approaches, we defined a melt pool angle *θ* associated with *θ'* in the single melt track (Figure 1b) and a columnar grain angle *α* (Figure 1a) to better describe the geometry of the melt pool and the related crystallographic orientation. The grain growth in sector BC (Figure 1a) can easily inherit the growth direction of grains in the previous layer (black arrows in Figure 2) or be hindered by the competitive grain growth mechanism.^[^ ^18^, ^34^ ^]^ This implies that their growth cannot dominate the crystallographic orientation of the entire sample. Thus, we chose sector CD to define these two angles. *θ* and *α* are the angles between the horizontal direction and the line CD and columnar grains, respectively (Figure 1a). Obviously, with increasing scanning speed, *θ* decreases and *α* respectively increases. The mean values of *θ* and *α*, averaged over 60 melt pools, are plotted in Figure 2f. These are, respectively, 56° ± 6° and 54° ± 6° for 600 mm s^−1^ (Figure 2b), 47° ± 5° and 62° ± 5° for 800 mm/s (Figure 2c), 39° ± 4° and 73° ± 4° for 1000 mm s^−1^ (Figure 2d), and 29°± 4° and 84° ± 5° for 1400 mm s^−1^ (Figure 2e). The linear dependence of *θ* and *α* on the scanning speed and the independent sum of *θ* and *α* (111° ± 5°) indicate that the crystallographic orientation can be tailored by the scanning speed. The growth direction and <001> orientation of the epitaxial columnar grains were not aligned with those of the TGD (Figure 1a). This is attributed to the preferential growth under a dynamic temperature gradient. At the beginning of the deposition, the dynamic evolution of the melt pool during solidification preferentially aligns the GDCG to the building direction, similar to that in additively manufactured metallic materials.^[^ ^18^, ^35^, ^36^ ^]^ As the deposition layer increases, preferential grains survive according to the competitive grain growth mechanism and epitaxial growth mechanism; continuous epitaxial growth occurs along a specific direction that deviates slightly from the TGD. In short, the MTCDS methodology was authenticated by the epitaxial growth of grains with different scanning speeds.

### 2.3 Crystallographic Orientation Evolution

**Figure 3:**
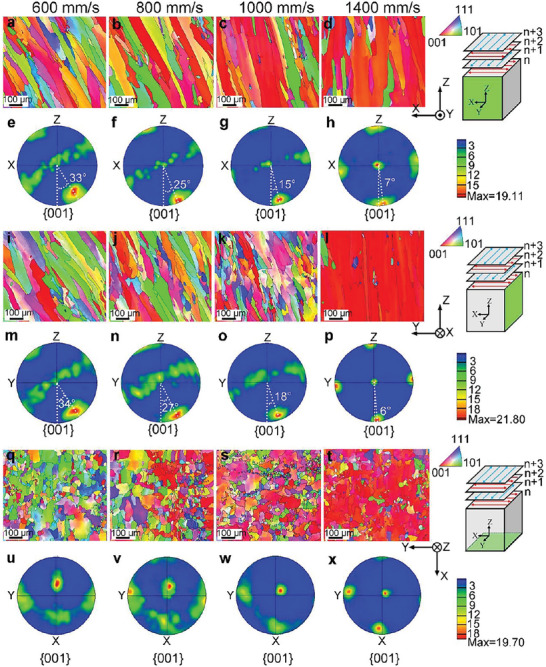
a‐d, i‐l, q‐t) Inverse pole figures (IPFs) and e‐h, m‐p, u‐x) corresponding {001} PFs of the a‐h) XZ‐plane, i‐p) YZ‐plane, and q‐x) XY‐plane of LPBF samples produced at scanning speeds of a, e, i, m, q, u) 600 mm s^−1^ , b, f, j, n, r, v) 800 mm/s, c, g, k, o, s, w) 1000 mm s^−1^ , and d, h, l, p, t, x) 1400 mm/s, respectively.

To elucidate the crystallographic orientation evolution at different scanning speeds, **Figure** **3** presents the inverse pole figures (IPFs) and corresponding {001} pole figures (PFs) of the XZ‐plane‐, YZ‐plane‐, and XY‐planes of LPBF samples produced at different scanning speeds. All PFs were derived by considering the upper hemisphere as the projection hemisphere. The four samples mainly consist of parallel columnar grains (Figure 3a–d,i–l), indicating that there is a strong <001> crystallographic orientation along their growth directions. The corresponding {001} PFs in the XZ‐plane (Figure 3e–h) and YZ‐plane (Figure 3m–p) show the evolution of the crystallographic orientation. At a scanning speed of 1400 mm s^−1^, the sample had a strong {100}<001> cubic texture (Figure 3h,p), whose three orthogonal <001> orientations were almost parallel to the X, Y, and Z directions. With a decrease in scanning speed, the intensity of the <001> fiber texture increased gradually, yet the {100}<001> cubic texture was still predominant (Figure 3e–h,m–p). Essentially, this texture evolution is related to GDCG depending on the melt pool angle *θ*. The GDCG gradually deviates from the Z‐axis with decreasing scanning speed (Figure 3e–h,m–p). The corresponding deviation angle between the <001> orientation and the building direction (*Z*‐axis) increased from 6–7° (Figure 3h,p) to 33–34° (Figure 3e,m), respectively, when the scanning speed decreased from 1400 to 600 mm s^−1^. This is in agreement with the variation in the columnar grain angle *α* with the scanning speed (Figures 2f and 3a–d,i–l). This variation tendency can also be confirmed by the IPFs (Figure 3q–t) and PFs (Figure 3u–x) in the XY plane. In detail, the red contrast of the <001> orientation gradually transforms into the green contrast of the <110> orientation with decreasing scanning speed. Thus, the texture analysis confirms the applicability of the MTCDS methodology for customizing the elastic modulus by tailoring the <001> orientation in additively manufactured parts. The wide range of deviation angles up to 28° (Figure 3e–h,m–p) provides a wide processing window in terms of the scanning speed (600–1400 mm/s) to obtain the <001> orientation along arbitrary 3D direction.

**Figure 4:**
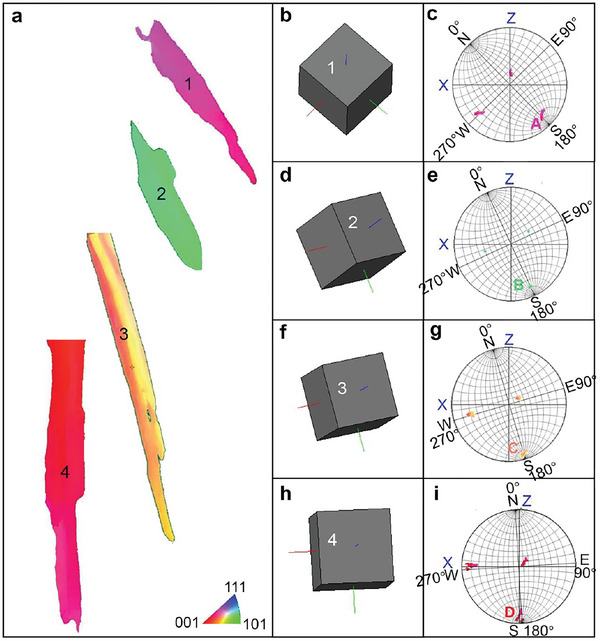
a) Typical grains 1, 2, 3, and 4 extracted respectively from the IPFs (Figure 3a–d) of the four samples. b,d,f,h) Corresponding 3D crystal orientation maps and c,e,g,i) {001} PFs projected on the XZ‐plane.

The pole clusters in the 4th quadrant in Figure 3e–h and m‐p deviate from the base circle. The corresponding deviation angle increased with decreasing scanning speed. A similar deviation was observed in a laser‐deposited Ni‐based superalloy^[^ ^37^ ^]^ and was proven by numerical simulation of arc‐welded stainless steel.^[^ ^20^ ^]^ To explain this deviation, **Figure** **4** shows the three‐dimensional crystal orientation maps and the {001} PFs of typical grains extracted from the IPFs of the four samples. Obviously, there is an orientation deviation angle between the growth direction of the columnar grains (green axis) and the XZ plane (Figure 4b,d,f,h). The orientation deviation angle, equal to the angle between poles A/B/C/D and pole S (Figure 4c,e,g,i), was determined to be approximately 14.2° for grain 1 (600 mm s^−1^), 10.1° for grain 2 (800 mm s^−1^), 6.3° for grain 3 (1000 mm s^−1^), and 3.2° for grain 4 (1400 mm s^−1^). The average orientation deviation angles of all columnar grains along the side (XZ/YZ‐plane) in Figure 3e–h,m–p were determined to be ≈11.3° at 600 mm s^−1^, 5.9° at 800 mm s^−1^, 6.1° at 1000 mm s^−1^, and 1.5° at 1400 mm s^−1^. The increased deviation angle agrees with the partial transition from the {100}<001> cubic texture to the <001> fiber texture (Figure 3e–h,m–p). In general, the easy‐growth direction (<001> orientation) always aligns closely with the TGD in the scanning strategy with 90° rotation. This cannot be satisfied by the {100}<001> cubic texture, which is formed by the <001> orientation parallel to the XY‐plane or YZ‐plane. To compromise with the TGD, the preferred <001> orientation is inclined to deviate from the XY‐plane or YZ‐plane. As such, deviations arise in Figures 3e–h,m–p and 4c,e,g,i. A similar scenario was presented in the numerical simulation of arc‐welded stainless steel.^[^ ^20^ ^]^ The underlying mechanism of crystallographic orientation deviation is attributed to the extent of the undercooled zone between the liquidus isotherm and the solidification front.

### 2.4 Customizing Elastic Modulus Along Arbitrary 3D Direction

**Figure 5:**
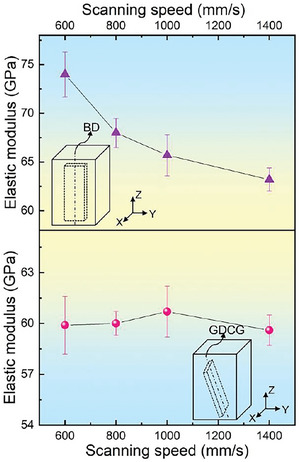
Elastic modulus of Ti‐35Nb‐7Zr‐5Ta samples obtained by LPBF, measured along building direction (BD) and GDCG, corresponding to <001> crystallographic orientation.

**Figure** **5** displays the elastic modulus of the LPBF‐processed Ti‐35Nb‐7Zr‐5Ta alloy, measured along the building direction (BD) and GDCG. BD is the direction of the *Z*‐axis and GDCG is the crystallographic <001> orientation, as shown in Figure 3e–h and m‐p. The elastic modulus along the GDCG is essentially the same, approximately 60 ± 1.2 GPa, which is far lower than that of LPBF Ti‐35Nb‐7Zr‐5Ta, i.e., 81 GPa.^[^ ^38^ ^]^ This value is slightly higher than that of cast Ti‐35Nb‐7Zr‐5Ta (55 GPa), which may result from a relatively high oxygen content (approximately 0.30 wt.%) of the LPBF‐processed samples. In contrast, the elastic modulus along the BD increases from 63.2 ± 1.2 GPa to 74 ± 2.3 GPa with decreasing scanning speed from 1400 to 600 mm s^−1^. As the four LPBF‐processed samples consisted of pure *β* phase (Figure S1, Supporting Information), the difference in the elastic modulus along the BD was mainly due to the crystallographic texture, as shown in Figures 3 and 4. Based on these results, it can be concluded that we can customize the <001> crystallographic orientation along an arbitrary 3D direction and resultant elastic modulus of the LPBF samples through the MTCDS methodology. This proves the feasibility of customizing elastic modulus values along a specific direction in biomedical implants, and provides a new method to minimize strain mismatch between implants and bone tissue (stress shielding).

**Figure 6:**
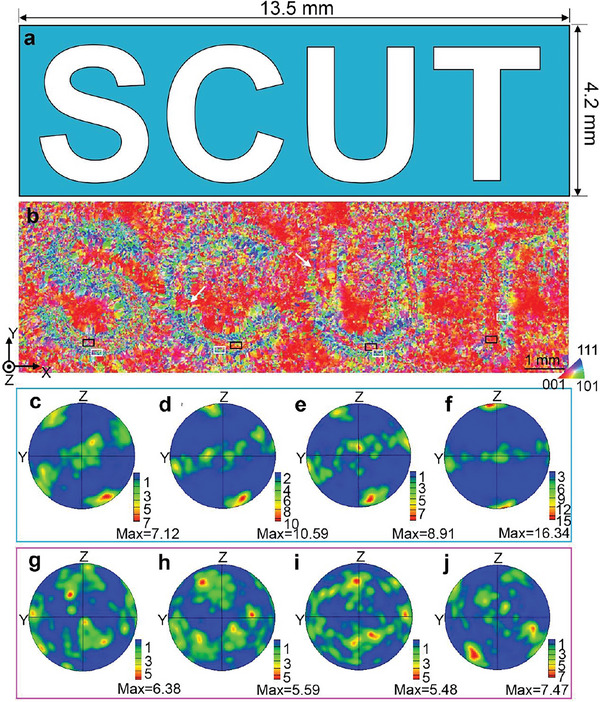
a,b) Letters S, C, U, and T produced by scanning speeds of 600, 800, 1000, and 1400 mm s^−1^, respectively, exhibiting different deviation angles relative to the building direction. The surrounding matrix is produced at the scanning speed of 1400 mm s^−1^. PFs of the inner and limbic regions of letters c, g) S, d, h) C, e, i) U, and f, j) T were projected on the YZ‐plane.

**Figure 7:**
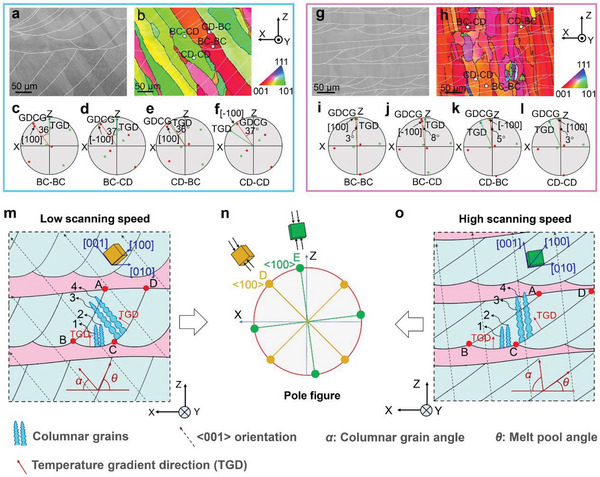
Cross‐section morphologies of melt pools on XZ‐plane produced at a) 600 mm s^−1^ and g) 1400 mm s^−1^. Corresponding b, h) IPFs and c‐f, i‐l) PFs of four typical micro‐areas produced at 600 mm s^−1^ and 1400 mm s^−1^ show the relationship between the GDCG (marked by red dotted arrows) and the TGD (marked by green arrows). m‐o) Diagrammatic sketches of directional solidification at m) low and o) high scanning and their n) PF.

**Figure** **6** presents four letters printed at different scanning speeds in a test area of 13.5 mm × 4.2 mm. The letters S, C, U, and T represent the South China University of Technology, with a height of 4 mm. The contrast of the columnar grains inside the four letters changed gradually from red (“T”) to green (“S”) as the scanning speed decreased (Figure 6b). In other words, the corresponding crystallographic orientation transformed from almost <001> (“T”) to <110> (“S”) relative to that of the BD. To confirm this result, Figure 6c–f shows the PFs of the inner region of the four letters projected onto the YZ‐plane. It is found that the deviation angle between the <001> orientation and the BD (*Z*‐axis) decreased from 31° (Figure 6c) to 7° (Figure 6f) with increasing scanning speed from 600 mm/s to 1400 mm/s, which is in good agreement with the results shown in Figure 3e–h,m–p. Compared with the same crystallographic orientation inside the letters, some grains along the letter boundary have non‐preferential orientations (indicated by white rectangles in Figure 6b, corresponding PFs in Figure 6g–j). Reasonably, this is caused by the complex temperature field at the letter boundary, which is the overlapping area of different sectors produced by different scanning speeds. This dilemma can be solved by multiple remelting and a reasonable overlapping ratio. This again shows that the MTCDS technique can be applied to program the <001> crystallographic orientation parallel to an arbitrary 3D direction in a single bulk component produced by AM.

## 3 Discussion

In this work, we established the MTCDS methodology, which can program a <001> crystallographic orientation along an arbitrary 3D direction in additively manufactured metallic materials by directional solidification, and thus epitaxial growth via precisely coupling multiple melt tracks with a tailored TGD (Figure 1, 2, 3, 4). MTCDS can customize the elastic modulus values of additively manufactured metallic materials (Figures 5 and 6). MTCDS is based on continuous epitaxial growth of a <001> crystallographic orientation along a specific direction by tailoring the LPBF parameters. Here, we discuss the influence of the laser parameters and their underlying mechanisms on crystallographic orientation evolution.

As shown in Figure 2, the melt pool angle *θ* and columnar grain angle *α* have opposite linear relationships with the scanning speed. Their detailed relationship can be determined by a simple mathematical calculation, and reveals the origin of the variation in crystallographic orientation. As shown in Figure 1a, the melt pool can be divided into three sectors (CD, BC, and CE). The growth of columnar grains in sector BC is hindered by a competition mechanism or inherits the crystallographic orientation of sector CD in the adjacent lower layer. Thus, CD in the current layer dominates the crystallographic orientation in the entire LPBF bulk material. In this case, the GDCG associated with *α* is mainly controlled by the temperature gradient of sector CD, which is related to *θ*. Based on the dependence of *ω* and *β*:^[^ ^25^ ^]^

(1) $$\tan\omega =\frac{l}{w}\tan\beta$$

where *ω* is the inclination angle between the substrate surface and the tangent of the boundary of the melt pool in the transverse section (XZ‐plane in Figure S2, Supporting Information), *w* is half the maximum width of a melt pool on the XZ plane, *l* is the length between the location of the maximum depth and the trailing point, *β* is the angle between the substrate surface and the tangent of the melt pool on the longitudinal section (YZ‐plane in Figure S2, Supporting Information). Due to the same direction of laser movement and the relatively large curvature radius of the CD segment, *θ* is approximately equal to *ω*. Furthermore, it is confirmed that *β* follows the function:^[^ ^39^ ^]^

(2) $$\tan\beta =\frac{V_{d}}{V_{b}}$$

where *V_d_* is the drill rate of a stationary laser for a given power, and *V_b_* is the laser scanning speed. *V_d_* can be calculated by using the following relation^[^ ^39^ ^]^:

(3) $$V_{d}=KA_{0}\frac{2P}{\pi D^{2}}$$

where *k* is a constant, *A*_0_ is the absorption, *P* is the laser power, and *D* is the laser beam spot size. Using Equation (1), (2), (3), *θ* can be determined as

(4) $$\tan\theta =\frac{l}{w}\frac{2kA_{0}P}{\pi D^{2}V_{b}}$$

Equation 4 indicates that the melt pool angle *θ* varies as a function of the LPBF processing parameters, especially the scanning speed *V_b_* and laser power. The value of $\frac{l}{w}$ has the same variation as *θ* with a specific LPBF processing parameter.^[^ ^20^ ^]^ From Eq. (4), *θ* decreases with increasing scanning speed *V_b_*, which is consistent with the results in Figure 2a,f. Consequently, a decrease in *θ* causes gradual turn of the TGD toward the *Z*‐axis. In other words, GDCG tends to align with the *Z*‐axis, leading to an increase in *α* (Figure 2f). It should be noted that the decreased melt pool angle *θ* is accompanied by an increased width‐to‐height ratio of the melt pools from 1.4 to 1.7, 2.3, and 3.9, respectively, with the increased scanning speeds from 600 to 800 mm s^−1^, 1000 mm s^−1^, and 1400 mm s^−1^, respectively, finally resulting in the crystallographic texture changes from <011> to <001> along the BD (Figure 3q–t). As reported in additively‐manufactured CoCrFeMnNi high‐entropy alloys,^[^ ^40^ ^]^ a melt pool with a small width‐to‐height ratio promotes the buildup of thermal gradients in a radial manner, leading to the <011> orientation along the BD. In contrast, in our present case, a melt pool with a large width‐to‐height ratio facilitates the alignment of maximum thermal gradient direction along the BD and consequently epitaxial growth across multiple layers with <001> orientation along the BD.

Equation 4 shows an inverse tangent relationship between *θ* and *V_b_*, which seems to be inconsistent with the linear relationship in Figure 2a,f. However, in this study, the inverse tangent relationship based on Equation 4 can be regarded as approximately linear (Figure S3, Supporting Information) when the scanning speed is between 600 and 1400 mm s^−1^. Thus, the results in Figure 2a,f agree with those determined from Equation 4. In addition, Figure 4 shows that the crystallographic orientation gradually deviates from the XZ‐plane with decreasing scanning speed. This can be attributed to two factors. The spatial distribution of the melt pool isotherm evolves with decreased scanning speed, transforming the melt pool from a teardrop shape to an elliptical shape.^[^ ^20^ ^]^ A decreased scanning speed can decrease the undercooling of metallic melts.^[^ ^41^ ^]^ This causes the front of the solid–liquid interface to approach the liquidus isotherm. These two factors result in increased curvature of the front of the solid–liquid interface and an increase in the angle *γ* (Figure S2, Supporting Information). Finally, a deviation in the crystallographic orientation is observed, as shown in Figure 4. The analysis confirms that the variation in the <001> crystallographic orientation originates from the melt pool angle *θ*. Likewise, the crystallographic texture evolution from the {100}<001> cubic texture to the <001> fiber texture is related to the melt pool geometry, the melt pool angle *θ*, and angle *γ*. The decreased scanning speed led to an increase in *θ* and *γ*, which caused the columnar grains to deviate from the XZ/YZ planes. Accordingly, it is necessary to rotate the <001> crystallographic orientation to adapt to the TGD, forming a <001>‐fiber texture. When the scanning speed increases, the columnar grains tend to grow along the BD and along the direction perpendicular to the scanning speed. In this case, two <001> crystallographic orientations perpendicular to each other are fixed; thus, it is easier to form a {100}<001> cubic texture.

It is known that LPBF is a track‐by‐track and layer‐by‐layer building technology in which a complex thermal history of a previous track and layer renders regular stacking between adjacent tracks and layers. Theoretically, it is feasible to enable columnar grains with specific orientations to grow epitaxially along the GDCG in the previous track and layer. This scenario can be easily achieved using the traditional directional solidification technology with a fixed heat flow direction. ^[^ ^42^ ^]^ Accordingly, the MTCDS method can accomplish similar directional solidification by regular stacking between adjacent tracks and layers. **Figure** **7** shows the OM photographs and IPFs of the LPBF‐processed samples produced at relatively low (600 mm/s) and high (1400 mm/s) scanning speeds. According to Figure 1a, melt pool can be divided into two sectors (CD and BC). Thus, the GDCG inside the two sectors depends on the grain orientation of the previous layer and track. All micro‐areas in the samples can be categorized into four combinations based on the assembly types of the CD and BC sectors: CD‐CD, CD‐BC, BC‐CD, and BC‐BC (Figure 7a,b,g,h, respectively). To determine the relationship between the GDCG and TGD in the sample produced at 600 mm/s, the TGD and six <001> crystallographic orientations were projected onto the XZ‐plane (Figure 7c–f). The TGD was approximately parallel to the XZ‐plane and perpendicular to the melt pool boundary (Figure 1a). The TGD at the bottom of the melt pool was parallel to the BD (Z‐axis). In the micro‐area with the CD‐CD combination, the [100] crystallographic orientation was determined to be the GDCG (Figure 7c) as it had the smallest angle. Similarly, the [−100], [100], and [−100] orientations were determined as GDCGs in the BC‐CD (Figure 7d), BC‐BC (Figure 7e), and CD‐BC (Figure 7f) micro‐areas, respectively. The inclination angles between the GDCG and Z‐axis were approximately 36.5° in the four typical micro‐areas, consistent with the corresponding angles, 36 ± 6°, in Figure 2f. Eventually, the entire sample achieved directional solidification. The LPBF sample produced at 1400 mm/s exhibited a similar directional solidification (Figure 7g–l). For visualization, Figure 7m–o shows diagrams of directional solidification in MTCDS. At a relatively low scanning speed (Figure 7m), the TGD in sector BC is almost parallel to the BD (Z‐axis); thus, few columnar grains (grains 1 and 2) grow along the TGD. However, their growth can be hindered by other columnar grains (grains 3 and 4) grown from sector CD. Subsequently, columnar grains in the next layer (sector DE) inherit the growth direction of grains 3 and 4 because the TGD matches the GDCG. Thus, they propagate through several layers and form a continuous <001> crystallographic orientation (Figure 7b). When the scanning speed increases, the melt pool angle *θ* decreases, and the TGD of both the CD and BC sectors is parallel to the BD. Thus, columnar grains grown from sectors CD and BC are compatible to form a continuous <001> crystallographic orientation (Figure 7h).

## 4 Conclusion

In the present work, we established an innovation methodology of MTCDS by stacking thin shells of 3D melt pool with the same temperature gradient direction, epitaxial growth of crystals between melt tracks, and ultimately directional solidification along a specific direction during AM. This MTCDS methodology can be used to program the <001> crystallographic orientation along an arbitrary 3D direction in additively manufactured metallic materials. Accordingly, the minimum elastic modulus along a specific direction was programmed in an additively manufactured beta‐type biomedical Ti‐35Nb‐7Zr‐5Ta alloy. It is expected that this methodology can be extended to tailor the properties of additively manufactured metallic materials which are sensitive to the <001> crystallographic orientation, such as for example creep resistance of nickel‐based superalloys, magnetic properties of Fe‐Si alloys, and corrosion resistance of Mg alloys. This study provides a universal methodology for the design of high‐performance metallic materials using AM.

## 5 Experimental Section

### Sample Preparation

Ingots with a nominal composition of beta‐type Ti‐35Nb‐7Zr‐5Ta (wt.%) alloy were used to fabricate a spherical powder with a size of D_50_ = 35.7 µm (Figure S4, Supporting Information) using Ar gas‐atomization equipment (AMC‐EIGA‐50, Beijing). The chemical composition of the powder analyzed by inductively coupled plasma atomic emission spectroscopy (iCAP PRO, USA) was found to be Ti‐35.5Nb‐7.6Zr‐4.2Ta‐0.22O (wt.%). The obtained powder was used for additive manufacturing of bulk samples with dimensions of 21 mm×21 mm×26 mm (block samples) and 20 mm×10 mm×4 mm (SCUT samples, Figure S4, Supporting Information) in EOS M290 laser powder bed fusion (LPBF) system. The LPBF unit is equipped with a 400‐W Yb:YAG laser (EOS GmbH, Krailling, Germany) with a wavelength of 1064 nm and a 100‐µm Gaussian spot diameter. The AM process was performed under a controlled atmosphere of high‐purity Ar gas. The substrate from Ti‐6Al‐4 V was preheated to 180 °C to reduce thermal stress. The block samples were fabricated with laser powers of 100–250 W, scanning speeds of 100–1800 mm s^−1^, hatch distances of 40 µm and 120 µm, and a fixed layer thickness of 30 µm. Bidirectional scanning was used with a rotation of 90° between layers (Figure S4, Supporting Information). For the SCUT sample, letters S, C, U, and T and their matrices were produced at optimized scanning speeds of 600, 800, 1000, and 1400 mm s^−1^, and a power of 250 W, a hatch distance of 40 µm, a layer thickness of 30 µm, and bidirectional scanning with a rotation of 90°.

### Material Characterization

Test samples with dimensions of 10 mm × 10 mm × 4 mm were cut out from the middle parts of the additively manufactured blocks using electrical discharge machining. Their XZ‐planes, YZ‐planes, and XY‐planes were ground with SiC emery papers up to P4000 grit and polished with a colloidal silica suspension with an average particle size of 0.04 µm. The XY‐plane perpendicular to the building direction was characterized by X‐ray diffraction (XRD; D/MAX‐2500/PC, Japan). The melt pool morphologies were observed using an optical microscope (OM, Leica DMC 4500, Germany). The IPFs and corresponding PFs of the XZ‐plane, YZ‐plane, and XY‐plane were determined using a scanning electron microscope (SEM, Leo 1530 Gemini, Zeiss) equipped with an electron backscatter diffraction (EBSD) system (e‐Flash, Bruker) at an accelerating voltage of 20 kV and a step interval of 1 µm. The XY‐plane of the LPBF SCUT sample was measured in several runs and montaged together to obtain the corresponding IPFs and PFs. EBSD maps were analyzed using professional analysis software (HKL Channel5, Oxford Instruments, UK) attached to the SEM. According to the ASTM E1876‐15 standard, the LPBF‐fabricated block samples were machined into dimensions of 25 mm × 10 mm × 2.5 mm to determine the elastic modulus using the impact excitation technique.

## Conflict of Interest

The authors declare no conflict of interest.

## Supporting information

Supporting Information

## Data Availability

The data that support the findings of this study are available from the corresponding author upon reasonable request.
