# Supplementary material for: Programming Crystallographic Orientation in Additive‐Manufactured Beta‐Type Titanium Alloy
Source: Adv Sci (Weinh). 2023 Jul 28;10(28):2302884. doi: 10.1002/advs.202302884 (PMC10558665; doi:10.1002/advs.202302884)
Supplement: Supplementary file 1 — Supporting Information [file ADVS-10-2302884-s001.pdf]

ADVANCED SCIENCE
Open Access

Supporting Information

for *Adv. Sci.*, DOI 10.1002/advs.202302884

Programming Crystallographic Orientation in Additive-Manufactured Beta-Type Titanium Alloy

Xuan Luo, Tao Song, Annett Gebert, Kai Neufeld, Ivan Kaban, Hongwei Ma, Weisi Cai, Haizhou Lu, Dongdong Li, Ning Li, Yuanyuan Li* and Chao Yang**

Supplementary Materials for

Programming crystallographic orientation in additive-manufactured beta-type titanium alloy

Xuan Luo[1, 2, 3], Tao Song[1], Annett Gebert[2, *], Kai Neufeld[2], Ivan Kaban[2], Hongwei Ma[1], Weisi Cai[1], Haizhou Lu[1], Dongdong Li[3], Ning Li[3], Yuanyuan Li[1, 3, *], Chao Yang[1, *]

[1] *National Engineering Research Center of Near-net-shape Forming for Metallic Materials, South China University of Technology, Guangzhou 510640, China*

[2] *Institute for Complex Materials, Leibniz IFW Dresden, Helmholtzstrasse 20, 01069 Dresden, Germany*

[3] *State Key Laboratory of Materials Processing and Die & Mould Technology, Huazhong University of Science and Technology, Wuhan, 430074, China*

---

[*] Corresponding author.

E-mail: A.Gebert@ifw-dresden.de (Annett Gebert); mehjli@scut.edu.cn (Yuanyuan Li); cyang@scut.edu.cn (Chao Yang), Tel & Fax: +86-20-87112111

Supplementary Figures:

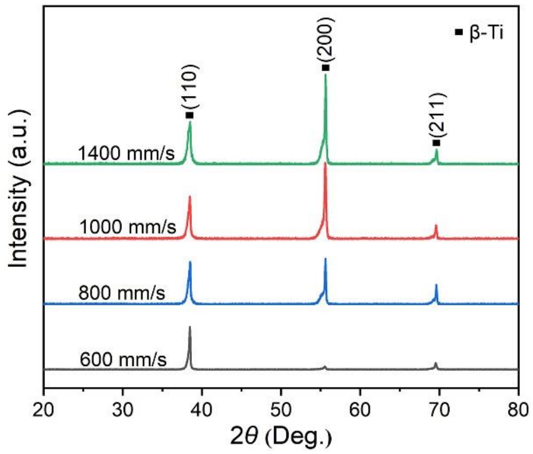

Fig. S1 XRD patterns of the XY-plane of the additively manufactured Ti-35Nb-7Zr-5Ta specimens with different scanning speeds.

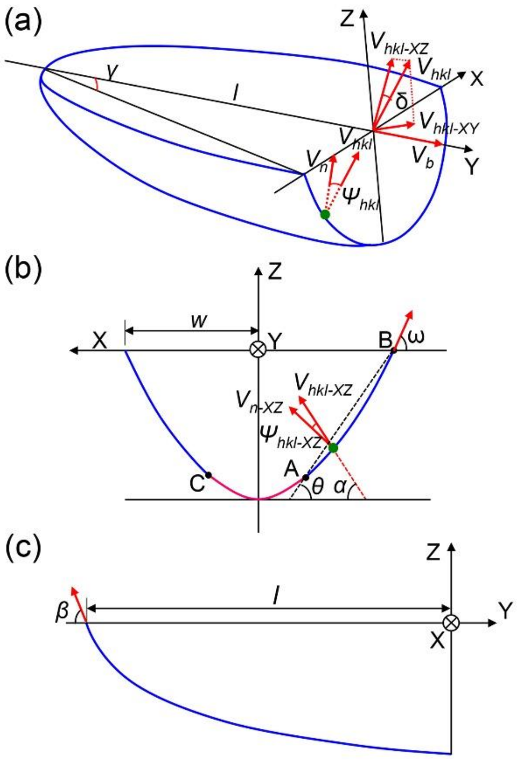

Fig. S2 (a) Schematic representation of the angular relationships between the scanning speed V_b, the solidification interface normal V_n, and the crystal growing direction V_{hkl}. Sections of the 3D melt pool in the XZ-plane (b) and XZ-plane (c). Ψ_{hkl} is the angle between the normal to the solidification interface and preferred [hkl] crystallographic direction. $V_{hkl\text{-}XY}$, $V_{hkl\text{-}XZ}$ is the the projection of V_{hkl} on the XY and XZ plane.

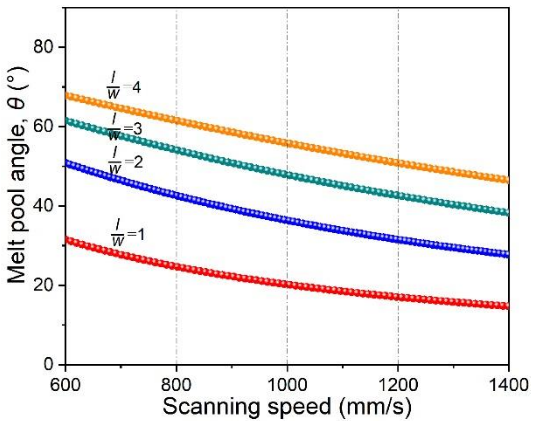

Fig. S3 The melt pool angle θ as a function of the scanning speed at different values of $\frac{l}{w}$.

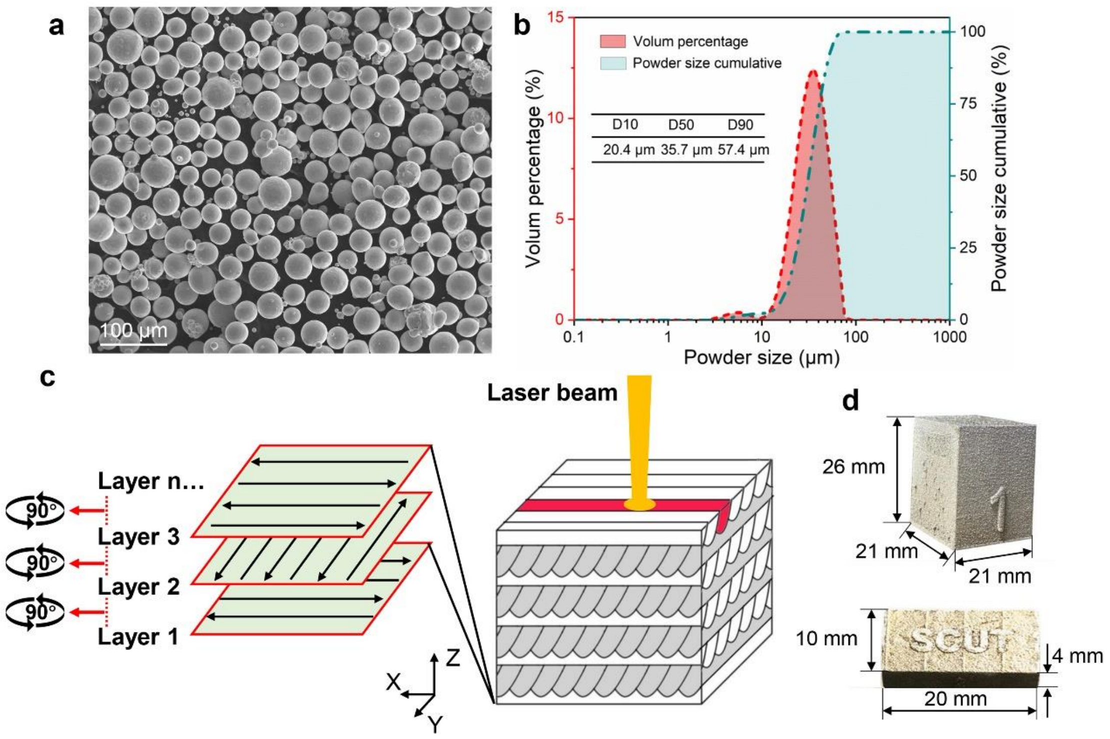

Fig. S4 (a) SEM image and (b) corresponding particle size distribution of the fabricated Ti-35Nb-7Zr-5Ta alloy powder. (c) Schematic representation of the scan strategy used in this work. (d) the additively manufactured block samples.
